# What Do We Think About Women Who Kill? Validation of the Attitudes Towards Female Perpetrators of Intimate Partner Homicide Scale

**DOI:** 10.1177/08862605251365649

**Published:** 2025-09-01

**Authors:** Sally Crosland, Calli Tzani, Maria Ioannou

**Affiliations:** 1 University of Huddersfield, UK

**Keywords:** women who kill, intimate partner homicide, female perpetrators, scale development, violent women

## Abstract

Although women who kill their intimate partners may be viewed in stereotypical ways, a method of measuring the extent of these stereotypical or biased attitudes about female perpetrators of intimate partner homicide did not previously exist. Prior beliefs may be utilised by jurors during decision-making alongside factual information presented during a trial, and characterisation of female defendants in the courtroom may have a potential influence on jury outcomes. To enable further exploration of the extent and impact of stereotypical beliefs amongst potential jurors, the attitudes towards female perpetrators of intimate partner homicide (AF-PIPH) scale was developed. Initial previous validation of the AF-PIPH scale via exploratory factor analysis suggested a 4-factor, 17-item structure. The aim of this study was to further test the structure of the scale via confirmatory factor analysis in a new participant pool. One hundred ninety jury-eligible participants aged between 18 and 75 were recruited to anonymously complete the AF-PIPH scale. During analysis, values were computed for the root mean square error of approximation (RMSEA), comparative fit index (CFI), Tucker-Lewis index (TLI), goodness of fit (GFI) and standardised root mean square residual (SRMR). After analysis, the four-factor structure was retained (χ^2^ = 152.53, *p* = .008, RMSEA = 0.043, CFI = 0.969, TLI = 0.963, SRMR = 0.057, GFI = 0.918) over an alternative three-factor model, with theoretical implications considered alongside measures of model fit. The AF-PIPH scale, therefore, has utility in identifying potential stereotypical or biased attitudes towards female homicide perpetrators, which may have benefits for the development of education and training programmes across sectors as well as within the legal system. Limitations are discussed, along with implications for jury selection, legal effectiveness reviews and potential contributions of the scale towards future research.

## Introduction

Female perpetrators of intimate partner homicide (IPH) are relatively understudied compared to their male counterparts. A contributing factor to the lack of focus on women who kill their intimate partners may be that the incidence of female-perpetrated IPH is relatively rare. In 2019, where the victim-perpetrator relationship was known, 2% of female-perpetrated IPH victims in the United States were classed as ‘boyfriends’ (Walker et al., 2023). In the United Kingdom, domestic homicide figures for the year ending March 2024 show 11 cases of female perpetration with a male victim, and 1 case where both perpetrator and victim were female, although data are not aggregated by specific victim-offender relationship (ONS, 2025). Motives for committing the homicide can vary, with prior incidence of domestic violence being a major contributor to perpetration of IPH by women against their male intimate partners (Swatt & He, 2016), although other motives may include jealousy, money (Gavin, 2015) or sexual proprietariness (Belknap et al., 2012). It is suggested that women who commit IPH are viewed stereotypically, even as evil or crazy (Walker et al., 2023). Pre-existing stereotypical beliefs influence jury decisions – if a female defendant is characterised as typically unfeminine, it is likely that she will receive a comparatively harsher sentence (Tillyer et al., 2015) compared to a defendant who is characterised as feminine. In addition, sentence length is also affected by motive, with motives deviating from traditional gender norms, such as for financial gain or as part of a sexual triangle, resulting in increased sentence severity in South Korea (Kim et al., 2018, p. 912). Stereotypical views or biases may manifest explicitly, where a bias is openly stated, or implicitly, where a bias is unconscious or implied (Dacon et al., 2021). Implicit biases may be positive or negative and involve associations based on stereotypes between social groups (Peters, 2019). In this instance, and specifically relating to the context of this research, biases regarding female IPH perpetrators may be both explicitly stated and implicit and unconscious. The effect these biases have within the criminal justice system includes typical expectations of the female role as being that of a caregiver, with disparities in sentencing when that role is seen to be violated (Russell & Torres, 2023). Minimisation of female violence is of concern, with benevolent sexism portraying female violence as lacking seriousness (Douglass, 2023). In addition, media portrayals of female IPH perpetrators are seen to attribute blame to their male victims (Hanson & Lysova, 2023). Conversely, women who seem to violate typical nurturing stereotypes may be viewed as doubly deviant (Mellor & Deering, 2010) and consequently attributed increased levels of culpability (Gavin, 2015). Women may also be viewed by the trope of mad, bad or sad, with the location of blame placed upon mental illness, prior experience of abuse, or upon the ‘bad’ woman herself, depending on her characterisation.

Despite this, there was previously no method to establish the extent of influence of specific stereotypical beliefs about female IPH perpetrators. To fill this gap, the attitudes towards female perpetrators of intimate partner homicide (AF-PIPH) scale (Crosland et al., 2023) was developed to measure pre-existing stereotypical beliefs about women who commit IPH. Initial validation of the scale was undertaken to finalise item questions and comprehension, with the items being reviewed by experts and public pools. The initial AF-PIPH scale was then subjected to exploratory factor analysis (EFA) in a sample of 103 jury-eligible participants from the United Kingdom, with a 4-factor, 17-item model structure suggested after analysis. The four factors reflected common stereotypes appearing across the literature, and were termed ‘The Other’, ‘The Victim’, ‘The Evil Woman’ and ‘The Nurturer’. The results of the EFA suggest that measurement of viewpoints relating to these stereotypes is possible. A short introduction to the factors follows below in Table 1.

**Table 1.** Introduction to the Four Factors of the AF-PIPH Scale, Previously Identified by EFA.

Factor	Description
The other	Female IPH perpetrators as other, different, wrong, or troublesome.
The victim	Female IPH perpetrators as acting as a last resort, battered women, not having a choice.
The evil woman	Female IPH perpetrators as crazy, mentally ill, evil, vile, should be publicly shamed, nasty, should be ashamed.
The nurturer	Female IPH perpetrators as naturally less violent than men, normally would not get violently angry, not in nature to kill.

*Note.* AF-PIPH = attitudes towards female perpetrators of intimate partner homicide; IPH = intimate partner homicide; EFA = exploratory factor analysis.

The implication of stereotypical beliefs becomes important when considering the decision-making process of juries in cases of female-perpetrated IPH. The significance of the role of prior belief in the deliberation process is emphasised in Pennington and Hastie’s (1986, 1988) Story Model of juror decision-making. Jury members tend to draw upon their previous knowledge and prior beliefs to aid in the decision process, alongside information gained from trial evidence (Pennington & Hastie, 1988). Gaps in evidence may be filled with a ‘story’, even though juror’s individual stories may not have a factual basis. These stories may have a cumulative effect on the eventual verdict (Conley & Conley, 2009) and have the potential to direct deliberations. The Story Model provides a comprehensive account of the process in which prior beliefs may affect jury verdicts, bringing together factual, evidential information and prior assumptions (Willmott et al., 2018, p. 27). Jurors construct their own individual stories as trials progress, weighing up ‘certainty principles’ of believability, uniqueness, coherence and coverage. Interestingly, and an assumption central to the Story Model, jurors who arrived at differing verdicts constructed varying stories (Pennington & Hastie, 1992, pp. 190–192). Individual interpretation of trial evidence may be affected by a juror’s life experiences and existing beliefs (Devine, 2012), with jurors filling in gaps in their constructed stories with intuition, common knowledge and ‘what they imagine to be the untold parts of the story’ (Ellison & Munro, 2015, p. 222).

In the United States, it is possible for attorneys to request the dismissal of jurors who may hold unfavourable views towards their case via peremptory challenges, or *voir dire*, in which potential jurors are questioned to both determine their fitness to serve, and to identify any potential biases (it is worth noting that the definition of *voir dire* differs between countries – in England, *voir dire* describes a process to determine admissibility of evidence before consideration by a jury). However, there is difficulty in identifying the possible stereotypical beliefs that may be held by an individual (Hastie, 1991), and lawyers may, in fact, end up relying on their own stereotypical views to inform their decisions regarding which jurors to retain. In fact, jurors with extreme beliefs may still be retained, although attorneys had a chance to remove them (Johnson & Haney, 1994). Although seemingly biased decisions to remove jurors may be challenged using the Batson framework (Batson v. Kentucky, 1986), the reliance on self-identification of biases means that attorneys are able to develop gender or race-neutral explanations for their decisions (Bornstein & Greene, 2017). In England, there is less scope for the removal of individuals from a jury via mechanisms such as peremptory challenge, although the Crown Prosecution Service allows for jury checks to be carried out to eliminate bias (Attorney General’s Office, 2012), involving a disclosure and barring service check on each juror to assess them against jury service criteria. There is also potential for further checks when necessary, including Special Branch and Security Services checks (Crown Prosecution Service, 2022). In addition, in security or terrorist cases, a juror may express extremely biased beliefs, which may exclude them from serving. Beyond this, however, no checks are conducted on any other sources. Therefore, it is entirely possible that jurors may hold stereotypical or biased beliefs that cannot be highlighted by these mechanisms. In cases of female-perpetrated IPH, the belief that a defendant is evil, unfeminine, or unrepentant may serve to direct the juror to process factual evidence in a way that serves to strengthen the story developed during the trial. Development of an attitude scale serves to identify how much of an effect these biases may have in a trial scenario, but also may be of use in education and training scenarios. Identification of stereotypical biases amongst those working alongside women who have killed their intimate partner can play a role in increasing awareness of the ways in which the prior experience of female IPH perpetrators may manifest in certain misunderstood behaviours, identifying opportunities for further education (Crosland et al., 2026). A salient example is the misunderstanding surrounding Battered Woman Syndrome (BWS; Walker, 2009), which may serve to sustain stereotypical views of women, specifically that of mental illness or passivity (Terrance et al., 2000). In addition, a defendant suffering from serious mental illness may not satisfy the insanity defence, but symptoms affecting behaviour may instead be attributed to personal flaws (Evans, 2022). Understanding the extent to which pre-existing views affect the ways in which we process and create stories around presented evidence may influence the presentation of expert evidence during a trial, with care taken to address erroneous biases that may exist in jury members. A tool allowing measurement of stereotypical beliefs may also be of use in training programmes. Within the criminal justice system, the ability to identify potential biases amongst training facilitators may serve to further enhance rehabilitative relationships and increase understanding – many female offenders have previously experienced abuse (Ministry of Justice, 2013), with many symptoms of trauma associated with long-term abuse being misunderstood or as a sign of culpability (Wistrich, 2022). Outside of the justice system, tools allowing measurement of biases in relation to female IPH perpetrators may be of use in future research concerning attitudes towards women who commit violent crime, jury decision-making and legal case reviews, amongst others.

## Aim

The current research aims to further test the internal model of the AF-PIPH scale structure via confirmatory factor analysis (CFA; Jöreskog, 1969), explore the internal consistency of the scale and discuss implications and further directions for future research.

## Method

Following initial validation of the AF-PIPH scale via EFA (Crosland et al., 2023), a 17-item, 4-factor structure was suggested. Further validation and exploration of the internal structure of the scale was required; therefore, data from a new participant sample were subjected to CFA to test the suggested model structure. CFA can be used to further develop models suggested by EFA, utilising separate participant groups (Wiebe et al., 2008). CFA tests the model fit of a hypothesised structure to observed data (Price, 2023), allowing testing of a hypothesised relationship between latent constructs and observed variables.

### Participants

Participants (*N* = 190) were recruited both from a sample of University Human and Health Sciences students via posters, internal communications and the university SONA system, and from the U.K. public via social media and word of mouth. Participants were screened to ensure that they met basic criteria for U.K. jury eligibility, namely that they were aged between 18 and 75 years of age, were registered to vote and had lived in the United Kingdom for a continuous period of 5 years since age 13. The participant screening procedure is outlined in the flowchart in Figure 1.

**Figure 1.**
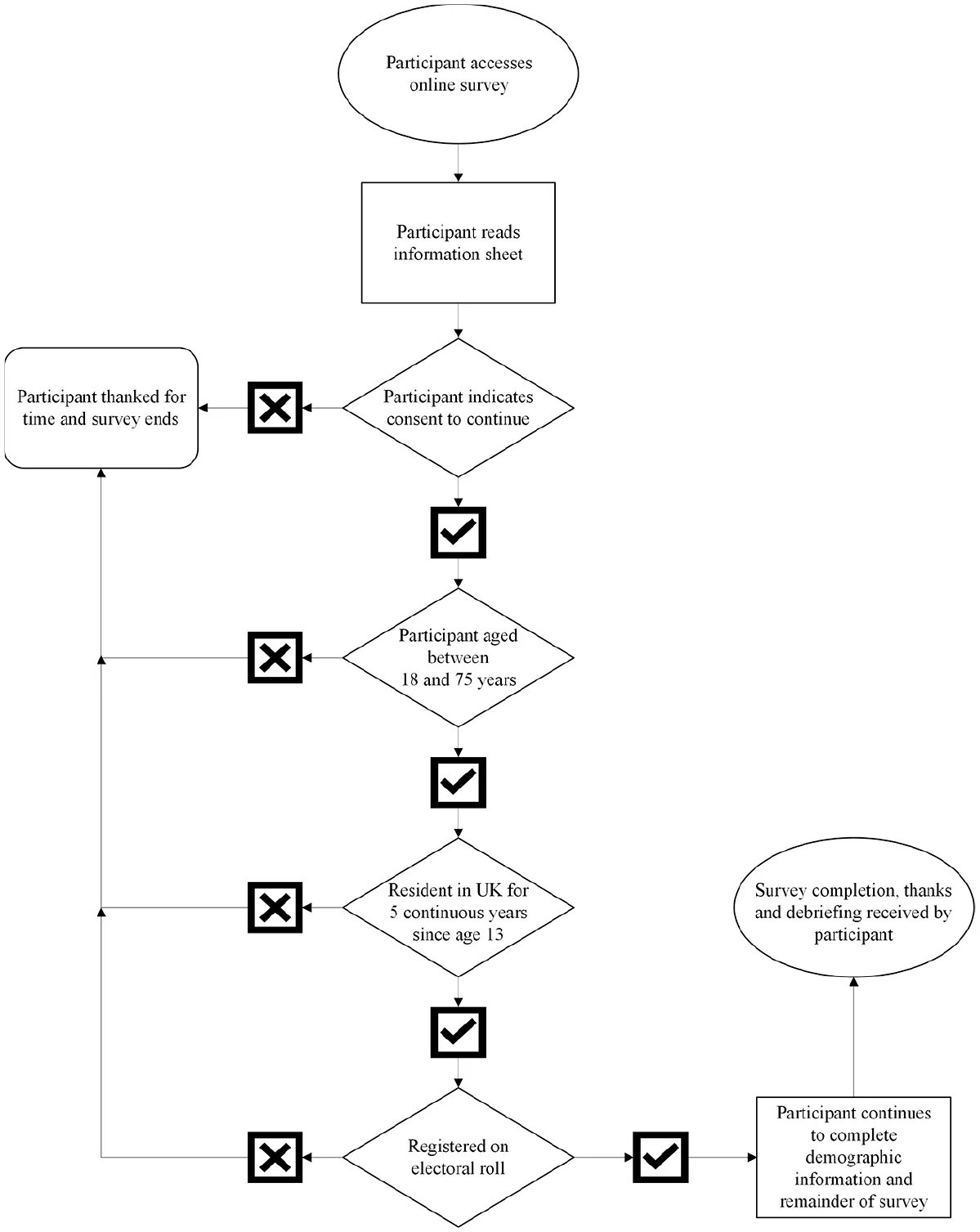
Flowchart of participant selection procedure.

An overview of participant characteristics can be found in Table 2.

**Table 2.** Participant Characteristics.

Category	Subcategory	Frequency	Percent (%)
Age	18–24	107	56.3
	25–34	28	14.7
	35–44	21	11.1
	45–54	23	12.1
	55–64	9	4.7
	65–74	1	0.5
	Prefer not to say	1	0.5
Gender	Male	26	13.7
	Female	155	81.6
	Non-binary	1	0.5
	Agender	1	0.5
	Prefer not to say	7	3.7
U.K. ethnic origin	White	155	81.6
	Black/African/Caribbean	5	2.6
	Asian – Indian, Pakistani, Bangladeshi, Chinese, or any other Asian background	27	14.2
	Mixed two or more ethnic groups	2	1.1
	Other – Arab or any others	1	0.5

### Methodology

A participant pool comprised of members of the public and university students anonymously completed the AF-PIPH scale online via the Qualtrics XM (Provo, UT) platform. After reading participant information and indicating their agreement to continue via a consent form, participants were asked to complete a number of demographic questions, followed by screening questions to ensure they met basic U.K. jury criteria. If they did not meet the jury criteria, the survey ended, ensuring all participants were representative of a U.K. jury. Once participants passed the screening questions, they were then asked to indicate their level of agreement with the series of 17 statements comprising the AF-PIPH scale. Participants rated their level of agreement with each statement on a five-item Likert scale, with points being *disagree strongly, disagree somewhat, neither agree nor disagree, agree somewhat* and *agree strongly*. Incomplete results were disregarded before analysis.

## Results

Kline (1993) suggests a participant/item ratio of at least 10:1 as a suitable sample size for CFA. As the scale structure for analysis comprised 17 item statements, this requirement was met. Data were analysed using SPSS AMOS v. 29 (IBM Corp), and there were no missing data. Suitability for factor analysis was confirmed by the Kaiser-Meyer-Olkin test = 0.880 and Bartlett’s test of sphericity χ^2^(136) = 1381.78, *p* < .001. Tests of normality were then performed on each item. Results indicated two items with skewness above 1; however, further tests of kurtosis revealed all items between values of −10 and +10, the indicator for normality (Collier, 2020). All items had kurtosis values <3.0, again indicating normal distribution of data (Westfall & Henning, 2013). As tests of kurtosis indicated normal distribution of data, 17 items were subjected to CFA using maximum likelihood estimation.

Although chi-square showed a poor model fit (χ^2^ = 152.53, *p* = .008) this result may be affected by the relatively small sample size (*N* = 190) (Rosseel, 2020), therefore other measures of fit were considered and indicated an appropriate model fit (RMSEA = 0.043, 90% CI [lower = 0.23, higher = 0.60], SRMR = 0.057, CFI = 0.969, TLI = 0.963). A three-factor model was also tested with three low-loading items removed; however, although indicators of model fit were slightly improved (see Table 3), there were issues with factor loading significance, internal consistency and a low number of items loading onto a factor. Therefore, it was decided to retain the four-factor model in this instance. Due to a relatively small sample size, the possibility of disregarding the four-factor solution may affect future exploration of the concepts suggested by an in-depth review of the literature and by EFA. In addition, results indicated a good model fit for the four-factor solution, with all items loading significantly onto their respective factors. Future exploration of factor structure is required, utilising larger sample sizes to further test validity and model fit.

**Table 3.** Results of Confirmatory Factor Analysis.

Model	χ^2^	*df*	χ^2^/*df*	CFI	TLI	GFI	RMSEA	RMSEA Lower CI	RMSEA Higher CI	SRMR	Factor Loadings			
											Evil Woman	Victim	Other	Nurturer
4 factor model	152.53	113	1.350	0.969	0.963	0.918	0.043	0.23	0.60	0.057	0.53–0.88	0.39–0.90	0.58–0.84	0.36–0.94
3 factor model	98.29	74	1.328	0.980	0.975	0.935	0.042	0.013	0.062	0.042	0.53–0.88	0.43–1.54	0.44–0.84	n/a

GFI= goodness of fit.

There is considerable debate surrounding acceptable values for factor loadings (Hair et al., 1998; Stevens, 1992); however, Tabachnick and Fidell (2007) suggest the following cutoff points for factor loadings: poor 0.32, fair 0.45, good 0.55, very good 0.63 and excellent 0.71.

Using the above suggested values, 13 items in the retained four-factor model showed good, very good, or excellent factor loadings, with 1 item having a fair factor loading (0.53) and three items having relatively low factor loadings of 0.39, 0.38 and 0.36, respectively. Despite these lower values, all items showed significant factor loadings. All items except three showed standardised regression weights above 0.5 see Table 4; Kline, 2015). In consideration of the items with a value below 0.5, after considering the theoretical implications of removal, it was decided to retain the items as indicators showed a good overall model fit, and all items showed significant loading.

**Table 4.** Standardised Regression Weights.

Factor	Item	Estimate	CRa	*p*
Evil woman				
	Kill_Crzy	0.732	—	***
	Pub_Shm	0.745	10.132	***
	Nst_Wom	0.880	12.046	***
	Mnt_Ill	0.528	7.082	***
	Kill_Evl	0.818	11.169	***
	Kill_Ashm	0.665	8.993	***
	Kill_Vile	0.787	10.725	***
Victim				
	Bat_Opt	0.899	—	***
	Dv_Chc	0.743	5.438	***
	Abu_Lres	0.394	4.333	***
Other				
	Diff_Kill	0.679	—	***
	Wrng_Kill	0.730	8.811	***
	Nt_Nrml	0.836	9.805	***
	Kill_Trbl	0.581	7.186	***
Nurturer				
	Norm_NViol	0.938	—	***
	Less_Voil	0.379	3.421	***
	Not_Ntr	0.360	3.341	***

*Note.* CR values unavailable for items with factor loadings set to 1 for analysis, denoted by —. CRa = critical ratio.

*** indicates *p* < .001.

The average variance extracted (AVE) was calculated as a measure of convergent validity for each factor. Composite reliability (CR) was then calculated as a measure of internal validity for each factor. AVE values ≥0.5 (Bagozzi & Yi, 1988) and CR values ≥0.7 (Nunnally & Bernstein, 1994) indicate acceptable validity. The factors ‘evil woman’, ‘victim’ and ‘other’ achieved convergent and internal validity (see Table 5). For the factor ‘nurturer’, AVE = 0.39 and CR = 0.61, indicating that internal validity was not achieved for this factor. Cronbach’s alpha (α; Cronbach, 1951) was also calculated as a measure of internal consistency for each factor. However, the utility of Cronbach’s alpha in structural equation modelling is debated, as it assumes equal factor loading. In cases where loadings are unequal, Cronbach’s alpha may underestimate the reliability of latent constructs (Anderson & Gerbing, 1988).

**Table 5.** Values of AVE, CR and Cronbach’s Alpha (α) for Individual Factors.

Factor	AVE	CR	α
Evil woman	0.56	0.90	.892
Victim	0.50	0.73	.705
Other	0.51	0.80	.799
Nurturer	0.39	0.61	.578

*Note.* AVE = average variance extracted; CR = Composite reliability.

### Covariance

Tests of covariance showed significant covariance between three of the factors: evil woman, other and nurturer, indicating an expected interrelation between constructs. One factor (victim) showed no covariance with the other three.

### Discriminant Validity

Correlations between factors were below .85, indicating achievement of discriminant validity (Kyriazos, 2018) and suggesting factors did not suffer from major problems of multicollinearity (Figure 2).

**Figure 2.**
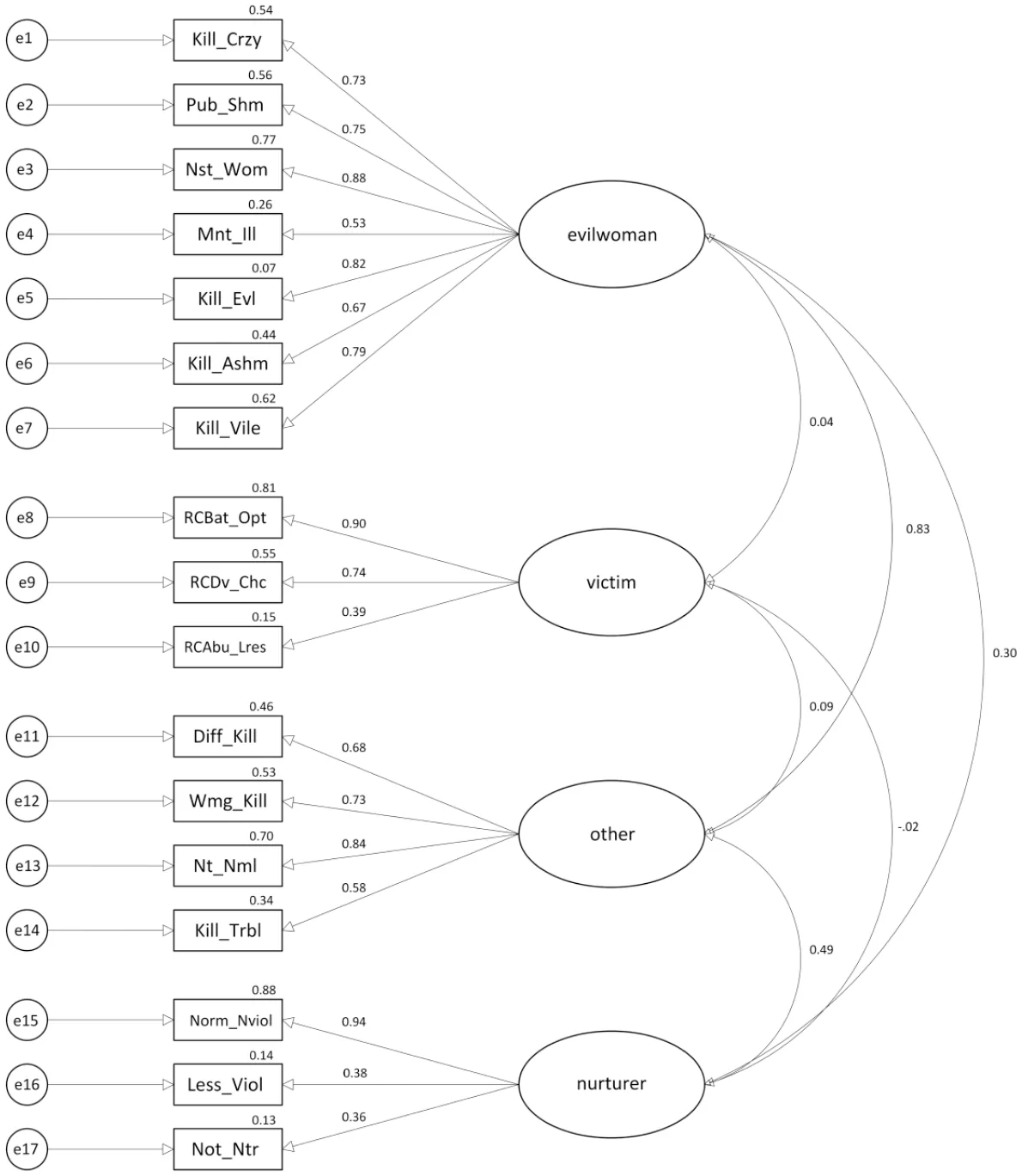
Path diagram.

### Further Estimation of Multivariate Models

As the participant sample was skewed towards white female respondents, additional multivariate analyses were performed to further explore demographic effects.

A multivariate analysis of variance (MANOVA) was performed, utilising the four factors (nurturer, evil woman, other and victim) as predictor variables and the AF-PIPH scale factor scores and total score as dependent variables. A one-sample Kolmogorov-Smirnov test showed that an assumption of normality was violated by four factors (nurturer *p* = <.001, victim *p* = <.001, other *p* = .013, evil woman *p* = .031), however the AF-PIPH total scores were normally distributed, *p* = .200. However, MANOVA is robust to violations of normality when sample sizes ≥25 (Geert van der Berg, n.d.). In this case, *n* = 190; therefore, it was decided to continue with MANOVA. Homogeneity of variance for each dependent variable was examined using Levene’s test: Nurturer, *p* = .184, Victim, *p* = .647, Other, *p* = .318, Evil Woman, *p* = .088, AF-PIPH total scores *p* = .382; therefore, homogeneity of variance is assumed. As group sizes were unequal, Pillai’s Trace is reported as it demonstrates robustness where MANOVA assumptions are violated (Bobbitt, 2021; Hair et al., 1998). For the overall AF-PIPH scale score, MANOVA showed a significant result for age category: Pillai’s Trace = 0.220, *F* (24, 652) = 1.583, *p* = .038, partial η^2^ = .055 and ethnicity: Pillai’s Trace = 0.169, *F* (16, 652) = 1.793, *p* = .028, partial η^2^ = .042. There was no significant result for gender: Pillai’s Trace = 0.114, *F* (12, 486) = 1.605, *p* = .087, partial η^2^ = .038.

Regarding each factor, tests of between-subjects effects highlighted significant effects of age category for ‘nurturer’: *F* (6, 163) = 2.237, *p* = .042, partial η^2^ = .076, ‘other’: *F* (6, 163) = 2.330, *p* = .035, partial η^2^ = .079 and ‘evil woman’: *F* (6, 163) = 2.300, *p* = .037, partial η^2^ = .078. In addition, there was a significant effect of U.K. ethnic origin on the factor ‘evil woman’: *F* (4, 163) = 3.485, *p* = .009, partial η^2^ = .079. Regarding gender, there was a significant effect of gender on the factor ‘other’: *F* (3, 163) = 4.691, *p* = .004, partial η^2^ = .079.

As some demographic groups contained single participants, post hoc tests could not be conducted for the variables age and ethnicity, as groups could not be omitted or combined. Data were combined for gender into four categories (male, female, non-binary and prefer not to say) to aid post hoc analysis; however, the non-binary category still only contained two participants, and as such, it would not be appropriate to draw solid conclusions regarding gender effects. However, Tukey’s honestly significant difference (HSD) test suggests potential gender effects for total AF-PIPH scores between male and non-binary participants (*p* = .011, 95% CI [3.55, 38.60]), and female and non-binary participants (*p* = .024 [1.78, 35.77]), accordingly, further research utilising larger samples across groups is recommended to further explore these potential differences and draw solid conclusions. However, and relevant for the sample utilised in this study, no significant gender effect was discovered between male and female participants for any factors or the AF-PIPH total score.

## Discussion

Results of CFA show a good model fit overall, with all factors apart from one displaying internal consistency at the required levels. An alternative, three-factor model was also subjected to CFA, and despite showing slightly improved model fit, measures of internal consistency and factor loadings were unsatisfactory; therefore, the four-factor model was retained in this instance. The 4-factor AF-PIPH scale showed significant loadings for all 17 items. As the overall model fit was good, in relation to the aims of this research, it is concluded that the AF-PIPH scale is reliable and has valid use in measuring attitudes towards female perpetrators of IPH. However, further discussion is warranted regarding individual factors within the model, and further exploration of the effects of gender, age and ethnicity would be of benefit in future stages of validation.

Initial validation of the AF-PIPH scale via EFA (Crosland et al., 2023) suggested four factors that contained items reflecting common stereotypes across the literature (evil woman, nurturer, other and victim). A woman who kills may be seen as evil, or conversely, may be seen as a victim with no other option. She may be expected to act in a nurturing way, judged harshly if her behaviour does not match up with expected stereotypes, or finally, she may be ostracised, seen as other and not ‘a woman like us’. As the factors measure aspects of the same theoretical underpinning, a level of correlation and covariance between factors was expected.

Three factors, ‘evil woman’, ‘other’ and ‘nurturer’, showed covariance; however, the factor ‘victim’ showed no covariance with the other factors. Relating to the theoretical underpinning of the scale, this was to be expected as items loading onto this factor were reverse-coded and relate to relatively positive attitudes towards women who are seen as victims due to the circumstances leading up to the homicide, whilst the other three factors measured similar concepts of othering, evil and the expectation of femininity. If a participant scores highly on one factor, theoretically, it would be expected that they would also score highly on related factors, but not on an unrelated factor. This seems to be the case in this instance, with the ‘victim’ factor including items that were reverse-coded and relate to positive stereotypes of female homicide perpetrators with specific experience of battering, abuse and domestic violence. The remaining three factors measure comparatively negative stereotypical views of female homicide perpetrators, relating to femininity, being seen as nurturing and the othering of women who fall outside of the feminine stereotype.

One factor, ‘nurturer’ did not achieve internal validity (AVE = 0.39, CR = 0.61). However, it was decided to retain the factor due to considerations of theoretical importance. There are multiple reasons that may affect the failure of this factor to achieve internal validity, with the limitations of sample size being a possibility. However, it is also feasible that the wording of the item questions was unclear and could be adapted – further research will be of benefit where larger samples can be recruited. The retention of the factor will allow for further exploration of the underlying concept that female IPH defendants, characterised as unfeminine, may receive a harsher sentence. This characterisation could be demonstrated in specific ways relating to nurturing behaviour, for example being described as a bad mother, a disobedient wife, or even as someone who does not easily show emotion – an important factor when considering manifestation of trauma responses due to prior abuse or trauma. For this reason, it was important to retain the factor despite the lack of internal validity, to contribute to the future potential of the scale. Although all factors showed acceptable discriminant validity (<0.85), the factors ‘evil woman’ and ‘other’ showed a higher correlation (.835). This is unsurprising. A woman seen as evil is, by definition, other. She exists outside of the normal behaviour expected of women. Gavin (2015) discusses the difficulty in comprehending the act of homicide by a woman against her intimate partner and the effect of BWS on the interpretation of female-perpetrated homicide. Misunderstanding of the experience of long-term abuse leads to the conclusion that the homicide must have been pre-planned, and therefore, the perpetrator is evil (p. 162).

As expected, the factor ‘victim’ has a lower correlation with the other factors, again echoing the reverse meaning of the items associated with this factor. With the blame located outside of the self, a woman seen as a victim is not responsible for her actions, and therefore less culpable. This contrasts with the attribution of blame suggested by the items associated with the three factors ‘other’, ‘nurturer’ and ‘evil woman’.

Three items showed low loadings onto their respective factors (see Table 4). An alternative, three-factor model was tested with these low-loading items removed; however, the overall three-factor model had issues with internal consistency and the number of items loading onto factors, despite showing slightly improved model fit when compared to the four-factor model. In light of this, it was decided to retain the low-loading items and the four-factor model, as the small improvement in model fit was counteracted by the poor internal consistency and item distribution across factors. In addition, the low-loading items showed previous high loading onto their respective factors during EFA. The items reflected important theoretical concepts, and so it was concluded that retention of the three low-loading items would be beneficial at this stage. Future testing and validation of the AF-PIPH scale will allow for further exploration. CFA is subject to several criticisms regarding the retention of specific numerical ‘cut off’ points that are used to denote model fit and the reliability of these specific indices (Prudon, 2015), however CFA remains of benefit, especially when developing measurement instruments (Jackson et al., 2009) such as the scale in question, although care must be taken not to rely entirely on the adoption of rule of thumb criteria as an absolute explanation of model fit. Hopwood and Donnellan (2010) identify limitations with the ‘golden rules’ of CFA model fit (p. 342) and identify issues with the adoption of stringent goodness-of-fit (GOF) criteria in the development of new instruments of measurement, where measures of external validity will not yet exist. Hopwood and Donnellan (2010) recommend several measures, including reporting of both EFA and CFA results, consideration of theoretical underpinnings and associated consequences of model modification, and the rejection of a ‘thumbs up or down’ decision (p. 343) of model fit. Marsh et al. (2004) highlight the limitations of Hu and Bentler’s (1999) work on GOF indexes, underlining Hu and Bentler’s (1999) original cautions that specific cutoff values may lack accuracy when considering differences in sample size or distributions and are not generalisable to all instances. Researchers are advised to keep in mind the idiosyncrasies of their particular study and seek to explain model misfit considering pertinent theoretical considerations (p. 340).

The structure of the AF-PIPH scale identifies differences between stereotypes in which female IPH perpetrators are seen as unfeminine, such as being seen as evil, and those in which the IPH perpetrator is seen to conform to an expected notion of femininity, for example being seen as a victim, supporting findings that suggest differences in sentencing severity when women are seen to deviate from expected feminine behaviour (Tillyer et al., 2015).

Belief in stereotypes, whether these biases form explicit views or are implicit, can serve to perpetuate the existing gender disparities in sentencing (Russell & Torres, 2023) and contribute to the continuing minimisation of female violence with implications not only for perpetrators, but also their victims. Therefore, work to identify biases and investigate the extent of their influence is important in beginning to tackle these implications. Confirmation bias, where individuals may misinterpret information in line with their existing beliefs and past experiences, has real implications in jury decision-making – as an example, a juror’s misunderstanding of symptom manifestation for individuals with diagnoses such as post-traumatic stress disorder may serve to increase understanding; conversely, it may also increase pathologisation. As Pennington and Hastie’s (1986, 1988) Story Model suggests, pre-existing biases influence the way in which jurors construct a story to fill in gaps in evidence during a trial. Therefore, biases, whether explicit or implicit, may influence how defendants are perceived by jurors. Stereotypes of ‘normal’ female behaviour are amplified by the media. Accounts of female-perpetrated homicide may be sensationalised and shared via social media channels at a rapid pace, contributing to broader societal biases and perpetuation of stereotypes (Russell & Torres, 2023). When identifying biases, it is important to acknowledge the background from which they may arise (National Academies of Sciences, Engineering, & Medicine, 2021). Implicit bias differs from explicit bias in that it is unconscious and may even contradict an individual’s conscious beliefs (Liu, 2023); therefore, addressing implicit biases requires policymakers to focus on social context to bring about change, tackling biases through mechanisms such as increased visibility (Payne & Vuletich, 2017), of which exploration of the effect of stereotypes is a preliminary step. The validation of the AF-PIPH scale contributes towards further research in this area, allowing further exploration of the extent to which stereotyping of female IPH defendants affects jury verdicts, as well as providing a tool to identify stereotypical biases amongst professionals working with women who have killed their intimate partners. Identification of misinformed or biased beliefs is an important step in the development of training programmes for staff and to identify areas where further education is needed – whether this is during an expert testimony or in a charity working alongside incarcerated women. Identification of stereotypical beliefs helps inform the development of new policies to raise visibility and tackle biases.

The identification of specific stereotypes has implications for jury decision-making. Jurors creating a story will look for factual information during a trial, aligning with their pre-existing beliefs (Pennington & Hastie, 1986, 1988), which may influence their overall verdict. Stereotypical characterisations of female IPH defendants during a trial may influence the formation of stories by jurors, adding to misunderstanding and perpetuating pre-existing biases. In countries such as the United States, where juror screening is permitted via peremptory challenge, the AF-PIPH scale could be utilised as part of a battery of tests comprising the pre-trial testing of potential jurors. In England, where pre-selection of jurors is limited, the AF-PIPH scale has utility in further research focusing on the experiences of female IPH defendants, specifically examining the effectiveness of legal defence, and where gender discrimination may influence tariff length (Justice for Women, n.d.).

The existence of phenomena such as the chivalry hypothesis and the evil woman hypothesis – where women conforming to a feminine stereotype are given comparatively lesser sentences than their male counterparts (chivalry) whereas women seen to commit the double deviance of committing a crime and also breaking out of the feminine stereotype (evil woman) are given comparatively harsher sentences – underline the need for further exploration in this area. The 4 identified factors in the AF-PIPH scale lend support to the continued existence of these hypotheses and provide a tool to examine the interaction between legal and extralegal factors in court outcomes (Tillyer et al., 2015).

## Limitations

This research had several limitations. Most importantly, the relatively small sample size may have influence on the model fit in this instance. Sample size was reduced by limitations on timescale and recruitment – many participants were recruited via convenience sample, with a relatively poor response to public recruitment attempts via social media in the available time period. Many indicators of model fit and validity may be affected by a smaller sample size, and therefore, further testing and cross-validation of the model is required with larger sample sizes in a new participant group to avoid sample-specific revisions (Cheung et al., 2024). The limitations of a smaller sample size may manifest in a model that demonstrates good fit overall but has issues of internal consistency in some areas (Stanley & Edwards, 2016), such as in this case. However, there is theoretical justification to retain the four-factor model here and a recognition of the limitations of a smaller sample in CFA.

Although guidelines regarding appropriate sample size indicate a participant: item ratio of 10:1 (Kline, 1993), it is wise to be cautious about the limitations of a smaller sample. However, disregarding the four-factor model would limit future opportunities to explore the predictive effect of the four main stereotypes suggested by the literature, and by EFA and CFA in the case of this scale. Failing to measure a concept known in the literature to be of importance and suggested during previous stages of validation may lead to the possibility of a type 1 error – premature rejection of a concept which may yet prove important. As stressed, further investigation is necessary.

Further exploration with a larger, gender balanced sample size may also show improved measures of internal consistency for the factor ‘nurturer’, which was the only factor that did not satisfy required levels of internal consistency. The issue of gender imbalance in the current sample meant that measures of gender invariance across model fit were not able to be explored in the current study, as the participant pool comprised predominantly female respondents. A gender balanced sample would be beneficial to aid exploration into gender differences, both in model fit and in future research utilising the scale. Although further multivariate tests suggest effects of both age category and U.K. ethnic origin on factor scores, further conclusions could not be drawn in this instance due to small group sizes. In this instance, there were no gender effects observed between male and female participants, which is salient due to the sample skew towards female respondents. Future stages of validation should further examine any potential demographic effects on both factor scores and overall scores of the AF-PIPH scale, and further testing of the scale with gender balanced samples, including non-binary individuals, is recommended as a future direction for exploration.

The current AF-PIPH scale focuses only on heterosexual relationships. Future iterations of the scale would benefit from revision to include sexual and gender-minority (SGM) relationship homicide scenarios, although due to the unique experiences and factors within non-heterosexual IPH situations (Anderson et al., 2023), development of unique scales to measure stereotyping in these situations may be of more use, as experiences cannot be generalised between heterosexual relationships and SGM relationships.

## Conclusion and Future Directions

Overall, a 4-factor, 17-item model was confirmed as the best fit via CFA, with overall good model fit, suggesting the validated AF-PIPH scale has utility in measuring AF-PIPH across four specific stereotypes – the nurturer, the other, the victim and the evil woman. Identification of the extent of belief in these stereotypes echoes the effect of the chivalry hypothesis and the evil woman hypothesis, and the scale will have utility in further exploration of these phenomena in relation to sentencing of female IPH perpetrators.

Future directions include measuring convergent and discriminant validity of the AF-PIPH scale by administering it alongside established scales measuring similar concepts, and further exploration of the effects of gender, U.K. ethnic origin and age. Another requirement is to explore the potential predictive validity of the AF-PIPH scale in mock jury trials of differing IPH scenarios. Exploration of the applicability of the AF-PIPH scale across countries is also an important future consideration. Variations in law, as well as cultural differences in the expected behaviour of women, may affect the efficacy of the AF-PIPH scale when used outside of the country of development. Testing of the scale in different populations will identify how far the underpinning theoretical considerations of the scale can be generalised. Development of a framework allowing identification of existing biases towards female IPH perpetrators would further add clarity. The scale also has utility in the identification of stereotypical attitudes with specific application to education and training for those involved with female homicide defendants, and additionally in academic research to further explore stereotyping in relation to female homicide perpetrators and jury decision-making.

## Data Availability

The datasets generated during and/or analysed during the current study are not publicly available due to institutional data protection policies.

## Appendix

The attitudes towards female perpetrators of intimate partner homicide (AF-PIPH) scale

17 items

1. A normal woman would not get violently angry.
2. Women are naturally less violent than men.
3. It is not in a woman’s nature to kill her partner.
4. A battered woman kills her partner as it is the only option.*
5. A woman who kills her partner after experiencing domestic abuse does not have a choice.*
6. An abused woman may kill her partner as a last resort.*
7. There must be something different about a woman that could kill her partner.
8. There is something wrong with women who kill their partners.
9. A woman who kills her partner is not a normal woman.
10. It is not surprising if a woman who has killed her partner has a history of being troublesome.
11. A woman who kills her partner is crazy.
12. A woman who kills her partner should be publicly shamed.
13. A woman who has killed her partner is a nasty woman.
14. A woman who kills her partner is mentally ill.
15. A woman who kills her partner is evil.
16. A woman who kills her partner should be ashamed.
17. A woman who has killed someone is vile.

* Reverse Coding:

*Items 4, 5 and 6 are reverse-coded.*

Scoring:

1 = Strongly disagree
2 = Slightly disagree
3 = Neither agree nor disagree
4 = Slightly agree
5 = Strongly agree
4 subscales

Evil Woman, Victim, Other, Nurturer.

### Nurturer

A normal woman would not get violently angry.

Women are naturally less violent than men.

It is not in a woman’s nature to kill her partner.

### Victim

A battered woman kills her partner as it is the only option.

A woman who kills her partner after experiencing domestic abuse does not have a choice.

An abused woman may kill her partner as a last resort.

### Other

There must be something different about a woman that could kill her partner.

There is something wrong with women who kill their partners.

A woman who kills her partner is not a normal woman.

It is not surprising if a woman who has killed her partner has a history of being troublesome.

### Evil Woman

A woman who kills her partner is crazy.

A woman who kills her partner should be publicly shamed.

A woman who has killed her partner is a nasty woman.

A woman who kills her partner is mentally ill.

A woman who kills her partner is evil.

A woman who kills her partner should be ashamed.

A woman who has killed someone is vile.
